# The characteristics of patients’ medical care, living will, and signs of death by age and the place of death: A cross-sectional study using a questionnaire survey targeting physicians with expertise in end-of-life care

**DOI:** 10.1371/journal.pone.0343868

**Published:** 2026-03-24

**Authors:** Mitsutaka Yakabe, Tatsuya Hosoi, Shoya Matsumoto, Shoho Miura, Kazuhiro Hoshi, Soichiro Iwao, Yoshihiro Kitamura, Masahiro Akishita, Sumito Ogawa

**Affiliations:** 1 Department of Geriatric Medicine, Graduate School of Medicine, The University of Tokyo, Bunkyo, Tokyo, Japan; 2 School of Engineering, The University of Tokyo, Bunkyo, Tokyo, Japan; 3 Japan Society For Dying With Dignity, Bunkyo-ku, Tokyo, Japan; 4 Nippon Medical School, Center for Medical Education, Bunkyo, Tokyo, Japan; 5 Tokyo Metropolitan Institute for Geriatrics and Gerontology, Itabashi-ku, Tokyo, Japan; University Hospital Cologne: Uniklinik Koln, GERMANY

## Abstract

**Objectives:**

Few studies have examined how age and place of death among end-of-life patients are associated with the medical care they receive and the signs of death. Therefore, we aimed to clarify the characteristics of medical care, the implementation of living wills, and the signs of death in end-of-life patients by administering a self-developed questionnaire to physicians with expertise in end-of-life care.

**Methods:**

Data were obtained through a web-based questionnaire administered to physicians registered with the Japan Society For Dying With Dignity. Each physician was asked to share data on a patient they had cared for, such as age, sex, place of death, presence of medical procedures and a living will, and signs of death.

**Results:**

In total, 437 patients, aged 79.4 ± 15.3 years, were analyzed. Pain control and palliative care were provided to 225 and 278 patients, respectively. Moreover, 172 patients possessed a living will. After adjusting for factors using logistic regression analysis, the odds ratios (ORs) for pain control and palliative care were significantly lower in the older age groups. The OR for pain control in nursing homes, compared with home, was 0.305 [95% confidence interval (CI), 0.081–0.920], and the OR for palliative care was 0.212 [95% CI, 0.073–0.544]. The ORs of living wills were significantly lower in patients aged ≥90 years (0.401 [95% CI, 0.199–0.800]) than in those aged 20–64 and in hospitals (0.494 [95% CI, 0.294–0.815]) than at home. Factor analysis of 18 signs of death identified three factors. Factor 1 reflected global terminal decline, and receipt of palliative care was significantly associated with the signs loading on factor 1.

**Conclusion:**

Provision of pain control, palliative care, and living wills differed by age group and place of death. Our findings suggest priorities for end-of-life care across settings and for representative studies.

## Introduction

Given the aging of the global population, mortality rates among older adults are predicted to increase in the future. Japan has already transitioned into a super-aging society, with senility ranking as the third leading cause of death, accounting for 11.4% of all deaths in 2022 [1].

In Japan, since 2019, the proportion of hospital deaths has been decreasing, while deaths at home and in nursing homes have been increasing [2]. Accordingly, there is a growing need to understand how clinical care is delivered across settings. Palliative care, rooted in the modern hospice movement, provides guidance for the care of seriously ill and dying patients to improve the quality of life of patients and their families [3]. A systematic review of the experiences of older adults nearing the end of life highlighted the physical discomfort associated with dying, with many experiencing potentially avoidable symptoms if care were improved [4].

Advance care planning (ACP) is a means of extending patients’ autonomy into periods when they may lack decision-making capacity and is defined as a process of discussion about goals of care and the recording of preferences for the care of patients who may lose decision-making or communication ability in the future [5]. Completion of a living will may occur as a result of ACP. A study reported that 36.7% of adults in the United States had completed an advance directive (AD) and 29.3% had a living will [6]. In Spain, the rate of AD completion is reported to be as low as 0.6%, indicating difficulties in promoting their use [7], whereas in Germany, approximately 44.8% of individuals aged 50 years and older have a living will [8], suggesting substantial cross-national differences. In Japan, ACP implementation has been reported at approximately 35–40% [9], but data on the specific situations in which a living will is obtained remain limited.

Patients with terminal illnesses manifest various signs. One of the most commonly observed signs is a decreased level of consciousness. In patients with cancer, impaired consciousness has been recognized as an early sign that may appear more than three days before death [10]. One study reported a temporal progression from decreased responsiveness to coma, which occurred in parallel with a cluster of highly specific physical findings during the last three days of life (e.g., mandibular breathing, peripheral cyanosis, loss of the radial pulse) [11]. A systematic review also reported that impaired consciousness, including confusion, was observed in approximately 50% of patients during the final two weeks of life [12].

Loss of appetite and inability to take oral nutrition are also frequently observed. In a prospective study conducted in an acute palliative care unit, decreased oral intake was the most common sign (60%) among patients assessed as being in their last two weeks of life, and it was accompanied by a progressive decline in consciousness and increasing care dependency [13]. In a cohort that included older adults without cancer, the median survival after the onset of markedly reduced oral intake was approximately 16.5 days, suggesting that this change may serve as a prognostic indicator about two weeks before death [14].

Other studies have highlighted pain and dyspnea as common indicators of impending death [15–17]. Delirium is also observed, often accompanied by restlessness and continuous physical movements [18–20].

Signs of death have been suggested to vary depending on the underlying cause of death. Most patients with cancer in the last 6 months of life had four or more impairments, with >40% experiencing serious pain, although the rates of depression and anxiety were only modest during the last 3 days of life [21]. When comparing the signs of death between patients with dementia and those with cancer, no significant difference was observed in the prevalence of dyspnea and agitation; however, patients with dementia exhibited significantly less pain than those with cancer [22]. A scoping review identified respiration with mandibular movement, particularly in the last 12 h of life, as a sign of impending death in both cancer and non-cancer patients [23]. Furthermore, signs of death may vary depending on the place of death. Patients who died at home were more likely to be fully alert shortly before death, less likely to be suffering from vomiting, incontinence, or bedsores, and less likely to have unrelieved physical distress [24]. A study that compared the characteristics of patients who died at home versus those who died in residential aged care facilities revealed that those who died at home tended to be younger, male, and die of cancer. They were more susceptible to nausea, whereas no significant differences were noted in the frequency of seven other symptoms [25].

Several studies on signs of death have collected information from individuals who witnessed patients’ final moments. Interviews with patients’ family members [26], questionnaire surveys of internists [27], and brainstorming sessions with caregivers [28] have been conducted to date.

The Japan Society For Dying With Dignity (JSDD), a public interest incorporated foundation since 1976, has promoted the concept of dignified death by issuing living wills and advocating for individuals’ right to live pleasantly and die peacefully (English site: https://songenshi-kyokai.or.jp/english). Physicians with expertise in end-of-life care are registered as “living will assisting physician” in JSDD. They have obtained written living wills and have cared for many patients at the end of their lives.

Thus, although evidence on the frequency and timing of common signs of death has accumulated, the associations of individual signs with factors such as age, place of death, and aspects of medical care remain poorly understood. Furthermore, few studies have examined how patient characteristics—such as the presence of a living will and the level of care—vary by age and place of death.

Therefore, this cross-sectional study had two main objectives. First, we quantified the associations of age group and place of death with three outcomes: pain control, palliative care, and the presence of a living will. Second, we examined the frequency and structure of various signs of death and to assess their associations with age, place of death, and aspects of medical care. To achieve these aims, we analyzed information obtained from physicians with substantial experience in end-of-life care, with the overall goal of generating actionable information for service planning across care settings.

## Methods

### Study design and settings

This study was a cross-sectional, web-based questionnaire survey conducted using Google Forms. The target population was physicians registered as “living will assisting physicians” in JSDD. Recruitment and data collection were conducted between August 5, 2021 (the study approval date) and March 31, 2023, with one reminder email sent during the recruitment period. This manuscript is reported in accordance with the Strengthening the Reporting of Observational Studies in Epidemiology (STROBE) statement [29].

### Participants

In 2021, we emailed a study invitation to the pre-existing mailing list used for routine communications with 2,000 “living will assisting physicians”; the list was not created for this study. The message was sent in a single batch to all 2,000 physicians. Those expressing willingness to participate were instructed to access the survey via the Google Forms link provided in the email.

Informed consent was obtained via Google Forms. Before responding to the questionnaire, all participants were required to review the study details presented on Google Forms and provide consent by selecting the “I agree” option. No name field was collected; therefore, responses were anonymous.

### Google Forms questionnaire and its development

After providing consent, participants were given the following instructions. “We would like to collect data on the signs of death in Japan and sincerely ask for your cooperation. Please share your valuable experience of witnessing signs that occur before a person’s death. Please answer about one patient you remember among those you cared for at the end of life within the past five years.” After this explanation, participants proceeded to the questionnaire shown in Table 1.

**Table 1:** The format of Google Form questions and answers.

No.	Question	Answer
1	How old was the patient at the time of death?	(his or her age)
2	What was the patient’s sex?	“male” or “female”
3	Where did the patient die?	“home,” “hospital,” or “others (free description)”
4	Was the patient receiving home oxygen therapy (HOT)? (Only if they died at home) If the answer is “Yes,” how long before death was the patient receiving HOT?	“Yes” or “No” “The day of death,” “1 day,””2 days,””3 days,””4 days,””5 days,””6 days,””1 week,””2 weeks,””3 weeks,””1 month,””2 months,””3 months,””4 months,””5 months,””6 months,””7 months,””8 months,””9 months,””10 months,””11months,””1 year,””2 years,””3 years,” or”4 years or over”
5	Was the patient receiving pain control? If the answer is “Yes,” how long before death was the patient receiving pain control?	“Yes” or “No” “The day of death,” “1 day,””2 days,””3 days,””4 days,””5 days,””6 days,””1 week,””2 weeks,””3 weeks,””1 month,””2 months,””3 months,””4 months,””5 months,””6 months,””7 months,””8 months,””9 months,””10 months,””11months,””1 year,””2 years,””3 years,” or”4 years or over”
6	Was the patient receiving palliative care? If the answer is “Yes,” how long before death was the patient receiving palliative care?	“Yes” or “No” “The day of death,” “1 day,””2 days,””3 days,””4 days,””5 days,””6 days,””1 week,””2 weeks,””3 weeks,””1 month,””2 months,””3 months,””4 months,””5 months,””6 months,””7 months,””8 months,””9 months,””10 months,””11months,””1 year,””2 years,””3 years,” or”4 years or over”
7	Did the patient have a living will?	“Yes” or “No”
8	What symptoms were frequently observed in the patient approximately one week prior to death? Choose all that apply.	#1. Lower level of consciousness and uttering incoherent statements. #2. Spending extended periods sleeping. #3. Experiencing difficulty consuming meals. #4. Struggling with drinking, leading to increased risk of aspiration. #5. Displaying agitation and heightened limb movement. #6. Decreased verbal communication, with diminished auditory clarity. #7. Appearing dazed and devoid of expression. #8. Experiencing urinary and fecal incontinence, with inability to reach the bathroom.
9	What symptoms were frequently observed in the patient approximately 48 hours prior to death? Choose all that apply.	#9. Diminished responsiveness to calls. #10. Impaired speech clarity #11. Exhibiting distress due to shallow breathing. #12. Difficulty in detecting pulse or experiencing a drop in blood pressure. #13. Limbs becoming cold. #14. Development of cyanosis on the face or extremities.
10	What symptoms were frequently observed in the patient approximately 12–24 hours prior to death? Choose all that apply.	#15. Sensation of warmth and reluctance to wear clothing. #16. Experiencing agitation or delirium #17. Slight limb movements despite being bedridden. #18. Tremors or shivering of coverings draped over the body.
11	Please feel free to write any comments based on your experience caring for this patient at the end of life (for example, messages you would like to share with future healthcare professionals or medical students, mindsets to keep in mind, challenges you noticed, or anything else).	(Free-text comments)

We developed the questionnaire through iterative discussions among the authors. Two authors (Soichiro Iwao and Yoshihiro Kitamura), who are physicians affiliated with JSDD and have extensive expertise in end-of-life care, led the selection and wording of key items. The remaining authors, although not end-of-life care specialists, are practicing physicians with experience in end-of-life care in home care and/or hospital settings and contributed to item review and refinement. As basic information, we included age and sex. To examine whether characteristics differ by setting, we added place of death. Because home oxygen therapy (HOT) is commonly used in home care, we also included its use. We included pain control, palliative care, and living will (as emphasized by JSDD), and we asked about the timing of their initiation. For the signs of death, we referred to B. Karnes’ booklet *Gone from My Sight* [30], which describes signs that are commonly observed three months to one month before death, two weeks to one week before death, a few days to a few hours before death, and in the last minutes before death. Using these time points as a reference, we specified three time points (one week before death, 48 hours before death, and 12–24 hours before death). For each time point, we listed signs extracted from the booklet and, in addition, signs proposed by the author group (all physicians experienced in end-of-life care) based on clinical experience. After repeated discussions, we finalized the items to be included in the questionnaire. Answers were mostly in multiple-choice format.

No inquiries were made about the patient’s personal information, such as name, date of birth, family, or residence. In the questionnaire, signs of death were assessed using three separate checklist questions corresponding to predefined time windows: approximately 1 week before death, approximately 48 hours before death, and approximately 12–24 hours before death. For each time window, participants were asked to select all signs they had observed in the patient during that window. The signs were numbered from 1 to 18 (symptoms 1–8 for 1 week before death, 9–14 for 48 hours before death, and 15–18 for 12–24 hours before death); the same set of 18 signs was not asked at every time window.

We added a free-text field at the end of the questionnaire. The prompt was as follows: “Please feel free to write any comments based on your experience caring for this patient at the end of life (for example, messages you would like to share with future healthcare professionals or medical students, mindsets to keep in mind, challenges you noticed, or anything else).” This was set to allow participants to provide contextual comments, but their responses were not collected for systematic qualitative analysis and were not used in any analyses in the present study; no separate qualitative analysis or publication is planned at present.

All entered data were used in the analysis, and no additional inclusion or exclusion criteria were applied. The questionnaire was not pilot tested before distribution.

### Data protection

After survey closure, responses were downloaded from Google Forms and de-identified, saved as a password-protected file on an encrypted USB device and stored in a locked drawer with access limited to the investigators. In accordance with the data management plan approved by the Research Ethics Committee of the University of Tokyo Hospital (2021150NI), the data will be deleted five years after study completion.

### The ethics statement

The study was approved by the Research Ethics Committee of the University of Tokyo Hospital (2021150NI) and adhered to the principles outlined in the Declaration of Helsinki.

As stated above, informed consent was obtained from the participants via Google Forms. The consent was recorded as part of the form data, along with their questionnaire responses. After downloading all data from Google Forms, it was confirmed that all participants had selected the “I agree” option.

This study was not registered, and no protocol was published in advance.

### Statistical analyses

After the response period, data were extracted from Google Forms and analyzed. One-way analysis of variance was performed to test for age differences between the groups. Chi-square tests were performed to assess differences in proportions across the groups.

Age was grouped a priori into four categories based on Japanese policy and geriatric conventions: < 65 years (non-geriatric adults), 65–74 years (young-old), 75–89 years, and ≥90 years (oldest-old). The cut-off at 75 years follows national administrative usage for older persons’ healthcare, and ≥90 years is frequently used to denote the oldest-old in geriatric research [31].

We pre-specified two logistic regression analyses aligned with our research questions. First, to identify factors associated with pain control, palliative care, and the presence of a living will, we fit multivariable logistic regression models with age group, sex, and place of death as predictors, reporting adjusted odds ratios (ORs) with 95% confidence intervals (CIs). Second, to examine how age, place of death, and medical care (pain control and palliative care) relate to each of the 18 signs of death, we fit separate logistic regression models for each sign. Because pain control and palliative care were strongly correlated, we checked for multicollinearity using the variance inflation factor (VIF). As both variables showed elevated generalized variance inflation factor (GVIF) values (>2), we included only palliative care as an explanatory variable in the multivariable models to avoid multicollinearity and improve interpretability.

We also performed an exploratory factor analysis of the 18 signs to explore latent domains and complement the sign-wise findings. The number of factors was determined by the Kaiser–Guttman rule (eigenvalues > 1.0) and inspection of the scree plot; when both solutions were plausible, clinical interpretability guided the final choice. Items with primary factor loadings ≥0.40 were used to label each factor.

*P*-values <0.05 were considered significant. All statistical analyses were performed using R version 3.3.3 (R Foundation for Statistical Computing, Vienna, Austria).

## Results

### Patient characteristics

**Fig 1:**
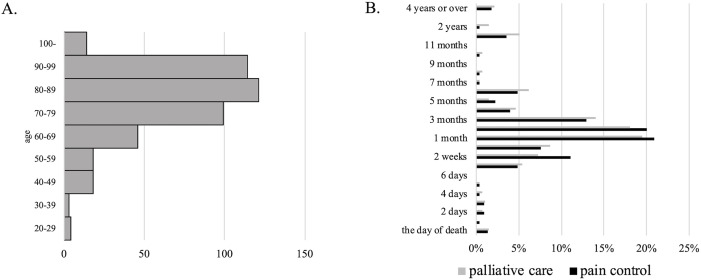
A. Age distribution of the patients. B. The distribution of onset times for pain control and palliative care.

Data on 440 patients were obtained through the Google Forms questionnaire. The patients’ ages ranged from infancy (<1 year) to 106 years. Patients who were minors (aged <20 years) at the time of death were excluded because their characteristics might differ from the majority, such as challenges in expressing their will and limited available facilities. After excluding the three patients, the remaining 437 were analyzed. The cohort included 210 male and 227 female patients, with an average age of 79.4 ± 15.3 years. The age distribution is shown in Fig 1A. Although the majority of the deceased patients were ≥65 years old, some were younger, with ages ranging from 21 to 106 years and a median age of 82 years.

The characteristics of the patients are shown in Table 2. Overall, HOT, pain control, and palliative care were provided in 148 (42.9%), 225 (52.7%), and 278 (64.8%) patients, respectively. Moreover, 172 (41.3%) patients had a living will. Regarding the signs of death, we assessed different pre-specified checklists for each time window. Among the eight signs assessed for approximately 1 week before death, symptoms 2, 3, 4, 6, 7, and 8 were most frequently reported. Among the six signs assessed for approximately 48 hours before death, symptoms 9, 10, 11, and 12 were most frequently reported. Of the four signs assessed for approximately 12–24 hours before death, symptom 17 was occasionally reported, whereas the other signs were rare.

**Table 2:** Characteristics of the patients (overall, and stratified by the place of death).

	Overall (n = 437)	Place of death				
		Home (n = 282)	Hospitals (n = 95)	Nursing homes (n = 30)	Other places (n = 25)	*p*-value
Age	79.4 ± 15.3	77.3 ± 15.7	77.9 ± 14.3	93.4 ± 4.0	89.8 ± 7.7	<0.001*
Sex						
Male	210 (48.1%)	136 (48.2%)	53 (55.8%)	8 (26.7%)	11 (44.0%)	0.047*
Female	227 (51.9%)	146 (51.8%)	42 (44.2%)	22 (73.3%)	14 (56.0%)	
HOT						
Yes	149 (42.9%)	127 (45.4%)	N/A	16 (59.3%)	5 (25.0%)	<0.001*
No	198 (57.1%)	153 (54.6%)	N/A	11 (40.7%)	15 (75.0%)	
Pain control						
Yes	225 (52.7%)	172 (61.2%)	45 (50.6%)	4 (13.8%)	4 (16.7%)	<0.001*
No	202 (47.3%)	109 (38.8%)	44 (49.4%)	25 (86.2%)	20 (83.3%)	
Palliative care						
Yes	278 (64.8%)	208 (74.6%)	52 (57.1%)	6 (20.7%)	11 (44.0%)	<0.001*
No	151 (35.2%)	71 (25.4%)	39 (42.9%)	23 (79.3%)	14 (56.0%)	
Living will						
Yes	172 (41.3%)	128 (48.1%)	29 (31.5%)	8 (26.7%)	7 (30.4%)	0.006*
No	244 (58.7%)	138 (51.9%)	63 (68.5%)	22 (73.3%)	16 (69.6%)	
Symptoms						
#1	89 (20.4%)	62 (22.0%)	17 (17.9%)	2 (6.7%)	8 (32.0%)	0.098
#2	289 (66.1%)	201 (71.3%)	52 (54.7%)	17 (56.7%)	19 (76.0%)	0.011*
#3	328 (75.1%)	224 (79.4%)	55 (57.9%)	26 (86.7%)	22 (88.0%)	<0.001*
#4	195 (44.6%)	134 (47.5%)	32 (33.7%)	11 (36.7%)	17 (68.0%)	0.008*
#5	25 (5.7%)	20 (7.1%)	3 (3.2%)	1 (3.3%)	1 (4.0%)	0.464
#6	227 (51.9%)	145 (51.4%)	48 (50.5%)	17 (56.7%)	16 (64.0%)	0.613
#7	200 (45.8%)	124 (44.4%)	44 (46.3%)	15 (50.0%)	16 (64.0%)	0.282
#8	210 (48.1%)	148 (52.5%)	42 (44.2%)	8 (26.7%)	12 (48.0%)	0.042*
#9	278 (63.6%)	183 (64.9%)	56 (58.9%)	21 (70.0%)	17 (68.0%)	0.617
#10	203 (46.5%)	147 (52.1%)	38 (40.0%)	8 (26.7%)	9 (36.0%)	0.012*
#11	182 (41.6%)	125 (44.3%)	40 (42.1%)	9 (30.0%)	8 (32.0%)	0.331
#12	169 (38.7%)	116 (41.1%)	36 (37.9%)	9 (30.0%)	7 (28.0%)	0.410
#13	145 (33.2%)	105 (37.2%)	22 (23.2%)	8 (26.7%)	9 (36.0%)	0.071
#14	92 (21.1%)	63 (22.3%)	14 (14.7%)	9 (30.0%)	5 (20.0%)	0.259
#15	15 (3.4%)	14 (5.0%)	1 (1.1%)	0 (0.0%)	0 (0.0%)	0.138
#16	23 (5.3%)	15 (5.3%)	5 (5.3%)	1 (3.3%)	2 (8.0%)	0.898
#17	119 (27.2%)	82 (29.1%)	25 (26.3%)	6 (20.0%)	5 (20.0%)	0.578
#18	43 (9.8%)	36 (12.8%)	4 (4.2%)	1 (3.3%)	2 (8.0%)	0.056

Age is shown as mean ± standard deviation.

Symptoms 1–18 are described in the Methods section. The number and the percentage of patients having each symptom are shown.

The sum does not necessarily match the total number because some patients had unanswered items.

The *p*-value was calculated by performing statistical analyses between the four groups of place of death. *p < 0.05 is considered significant.

### Correlation between pain control and palliative care

Spearman’s correlation coefficient between pain control and palliative care (presence/absence) was 0.711 (p < 0.001), indicating a significant positive correlation. In addition, the distribution of onset times for both was similar, occurring the most commonly 1–3 months before death (Fig 1B). Spearman’s correlation coefficient for the onset times was 0.728 (p < 0.001), indicating statistical significance.

### Characteristics of patients based on the place of death

To determine differences in patient characteristics, patients were analyzed according to the place of death (Table 2). Among the 437 patients, the most frequent place of death was at home (n = 282, 64.5%), followed by hospitals (n = 95, 21.7%). Other responses (free description) included facilities such as private nursing homes, specialized nursing homes, group homes, geriatric health services facilities, and small-scale multifunctional care facilities. Among those, 30 (6.9%) patients residing in private or specialized nursing homes were categorized as the nursing homes group. In addition, 25 (5.7%) patients who did not fit into any of these groups were defined as the other place group, and 5 (1.1%) patients had an unspecified place of death.

The average age of those in the nursing home group was 93.4 ± 4.0 years, significantly higher than those in the home (77.3 ± 15.7 years) and hospital (77.9 ± 14.3) groups. In the home group, 61.2% and 74.6% of the patients received pain control and palliative care, respectively, which were significantly higher than those in the hospital (50.6% and 57.1%, respectively) and nursing homes (13.8% and 20.7%, respectively) groups. In the home group, 48.1% of the patients had a living will significantly higher than that in the hospital (31.5%) and nursing homes (26.7%) groups.

### Characteristics of patients based on the age at death

Whether characteristics varied by age at death was also examined. The patients were categorized into four age groups: aged 20–64 (n = 61), 65–74 (n = 79), 75–89 (n = 169), and ≥90 years (n = 128).

The characteristics of these groups are shown in Table 3. Significant differences in the sex ratio were found between the groups: the proportion of female patients was higher in the groups aged 20–64 and >90 years, and that of male patients was higher in the groups aged 65–74 and 75–89 years. The younger the age at death, the higher the proportion receiving pain control and palliative care, that is, 85.0% and 95.0% for those aged 20–64 years and 16.1% and 35.4% for those aged ≥90 years, respectively. Moreover, in the group aged >90 years, 27.9% had a living will, which was significantly lower than the rates in other groups. More patients aged 20–64 years were using HOT; however, the difference was not significant compared with other age groups.

**Table 3:** Characteristics of the patients stratified by age.

	Age (years old)^†^				*p*-value
	20–64 (n = 61)	65–74 (n = 79)	75–89 (n = 169)	90 – (n = 128)	
Age	50.6 ± 11.1	70.1 ± 2.9	82.5 ± 4.3	94.6 ± 3.7	–
Sex					
Male	19 (31.3%)	47 (59.5%)	108 (63.9%)	36 (28.1%)	<0.001*
Female	42 (68.9%)	32 (40.5%)	61 (36.1%)	92 (71.9%)	
Place of death					
Home	48 (80.0%)	53 (67.1%)	118 (70.2%)	63 (50.4%)	<0.001*
Hospitals	12 (20.0%)	25 (31.6%)	37 (22.0%)	21 (16.8%)	
Nursing homes	0 (0.0%)	0 (0.0%)	5 (3.0%)	25 (20.0%)	
Other places	0 (0.0%)	1 (1.3%)	8 (4.8%)	16 (12.8%)	
HOT					
Yes	28 (57.1%)	24 (44.4%)	61 (43.4%)	36 (35.0%)	0.080
No	21 (42.9%)	30 (55.6%)	80 (56.7%)	67 (65.0%)	
Pain control					
Yes	51 (85.0%)	49 (64.5%)	105 (62.9%)	20 (16.1%)	<0.001*
No	9 (15.0%)	27 (35.5%)	62 (37.1%)	104 (83.9%)	
Palliative care					
Yes	57 (95.0%)	58 (77.3%)	118 (70.7%)	45 (35.4%)	<0.001*
No	3 (5.0%)	17 (22.7%)	49 (29.3%)	82 (64.6%)	
Living will					
Yes	30 (51.7%)	34 (45.3%)	74 (46.0%)	34 (27.9%)	0.003*
No	28 (48.3%)	41 (54.7%)	87 (54.0%)	88 (72.1%)	
Symptoms					
#1	19 (31.1%)	19 (24.1%)	32 (18.9%)	19 (4.8%)	0.054
#2	47 (77.0%)	51 (64.6%)	113 (66.9%)	78 (60.9%)	0.178
#3	50 (82.0%)	60 (75.9%)	129 (76.3%)	89 (69.5%)	0.281
#4	31 (50.8%)	36 (45.6%)	70 (41.4%)	58 (45.3%)	0.636
#5	9 (14.8%)	3 (3.8%)	9 (5.3%)	4 (3.1%)	0.010*
#6	35 (57.4%)	33 (41.8%)	94 (55.6%)	65 (50.8%)	0.173
#7	34 (55.7%)	39 (49.4%)	69 (40.8%)	58 (45.3%)	0.210
#8	37 (60.7%)	35 (44.3%)	92 (54.4%)	46 (35.9%)	0.002*
#9	47 (77.0%)	48 (60.8%)	102 (60.4%)	81 (63.3%)	0.121
#10	39 (63.9%)	44 (55.7%)	79 (46.7%)	41 (32.0%)	<0.001*
#11	31 (50.8%)	35 (44.3%)	76 (45.0%)	40 (31.2%)	0.032*
#12	32 (52.5%)	30 (38.0%)	65 (38.5%)	42 (32.8%)	0.080
#13	25 (41.0%)	26 (32.9%)	59 (34.9%)	35 (27.3%)	0.276
#14	18 (29.5%)	15 (19.0%)	34 (20.1%)	25 (19.5%)	0.377
#15	5 (8.2%)	5 (6.3%)	5 (3.0%)	0 (0.0%)	0.013*
#16	7 (11.5%)	6 (7.6%)	7 (4.1%)	3 (2.3%)	0.042*
#17	19 (31.1%)	23 (29.1%)	46 (27.2%)	31 (24.2%)	0.753
#18	10 (16.4%)	9 (11.4%)	18 (10.7%)	6 (4.7%)	0.068

†Patient below 20 years old were excluded.

Age is shown as mean ± standard deviation.

*p*-value was calculated by performing statistical analyses between the four groups of age. *p < 0.05 is considered significant.

In the Symptoms column, the number and the percentage of patients having each symptom are shown.

The sum does not necessarily match the total number because some patients had unanswered items.

### Factors determining pain control, palliative care, and living will

A logistic regression analysis was performed to evaluate factors that affect pain control, palliative care, and living will. Patients with unspecified place of death were excluded, leaving 432 patients for analysis. Each model was adjusted for age group, sex, and place of death (reference categories: 20–64 years for age; home for place of death; male for sex).

The results of logistic regression analysis are presented in Table 4. We fit separate multivariable logistic regression models for three pre-specified outcomes (pain control, palliative care, and living will). Because these outcomes represent distinct constructs and each model addresses a separate research question, we did not apply multiplicity adjustments across the three analyses. In line with recommendations for exploratory clinical research, we emphasize effect sizes and 95% CI rather than relying solely on p-values. The p-values presented should therefore be interpreted as descriptive.

**Table 4:** Logistic regression analysis to evaluate factors on the state of pain control, palliative care, and living will.

	Pain control		Palliative care		Living will	
Variable	ORs [95% CI]	*p*-value	ORs [95% CI]	*p*-value	ORs [95% CI]	*p*-value
Sex (female)	1.188 [0.742–1.920]	0.476	1.104 [0.682–1.803]	0.690	1.100 [0.716–1.694]	0.664
Age^†^						
20-64	1.000	–	1.000	–	1.000	–
65-74	0.358 [0.144–0.828]	0.020*	0.207 [0.046–0.673]	0.017*	0.828 [0.405–1.687]	0.603
75-89	0.358 [0.152–0.766]	0.012*	0.147 [0.034–0.435]	0.002*	0.837 [0.443–1.578]	0.581
90-	0.045 [0.018–0.105]	<0.001*	0.039 [0.009–0.117]	<0.001*	0.401 [0.199–0.800]	0.010*
Place of death^††^						
Home	1.000	–	1.000	–	1.000	–
Hospitals	0.603 [0.354–1.028]	0.062	0.422 [0.245–0.724]	0.002*	0.494 [0.294–0.815]	0.007*
Nursing homes	0.305 [0.081–0.920]	0.050*	0.212 [0.073–0.544]	0.002*	0.611 [0.236–1.465]	0.285
Other places	0.237 [0.063–0.719]	0.017*	0.529 [0.213–1.292]	0.162	0.632 [0.230–1.588]	0.344

† Those aged 20–64 = 1.

†† Home = 1.

CI: confidence interval. *p < 0.05 is considered significant.

After adjustment for age group (20–64 [reference], 65–74, 75–89, ≥ 90 years), sex (male [reference], female), and place of death (home [reference], hospital, nursing home, other places), the odds of pain control were lower in ages 65–74 years (OR 0.358, 95% CI 0.144–0.828), 75–89 years (OR 0.358, 95% CI 0.152–0.766), and >90 years (OR 0.045, 95% CI 0.018–0.105) compared with 20–64. By place of death, compared with home, the odds of pain control were lower in nursing homes (OR 0.305, 95% CI 0.081–0.920) and in other places (OR 0.237, 95% CI 0.063–0.719). For palliative care, compared with 20–64 years, the odds were lower in ages 65–74 years (OR 0.207, 95% CI 0.046–0.673), 75–89 years (OR 0.147, 95% CI 0.034–0.435), and >90 years (OR 0.039, 95% CI 0.009–0.117). Compared with home, the odds were also lower in hospitals (OR 0.422, 95% CI 0.245–0.724) and in nursing homes (OR 0.212, 95% CI 0.073–0.544). The ORs for a living will were significantly lower in the group aged >90 years than that aged 20–64 years (OR 0.401, 95% CI 0.199–0.800) and in the hospital group than in the home group (OR 0.494, 95% CI 0.294–0.815). Sex difference was not significant in any of the analyses.

### Factor analysis and logistic regression for signs of death

To identify common factors that influence symptoms 1–18, factor analyses on the questionnaire results were conducted. First, all 18 symptoms were analyzed. Three factors (1–3) were determined based on eigenvalues >1.0 and the scree plot (Table 5). Symptoms 2, 3, 4, 6, 7, 8, 9, 10, and 17 were primarily influenced by factor 1, 13 and 14 were mainly influenced by factor 2, and 11 and 12 were moderately influenced by factors 1 and 2. Symptoms 1, 5, 16, and 18 were primarily influenced by factor 3. Symptom 15 was not significantly influenced by factors 1, 2, or 3. The variance proportions of factors 1, 2, and 3 were 0.202, 0.072, and 0.056, respectively, with a cumulative variance of 0.329. The three factors represent clinically interpretable latent constructs derived from the 18 end-of-life signs. Factor 1 reflects global terminal decline (neurocognitive–functional deterioration), with high loadings for decreased consciousness and communication, poor oral intake, incontinence, and diminished responsiveness. Factor 2 captures peripheral circulatory and skin changes, including cold extremities and cyanosis. Factor 3 represents terminal agitation or delirium with motor restlessness, including agitation, incoherent utterances, delirium-like behaviors, and shivering of coverings. These factors reflect clinically recognizable domains frequently observed in the final stage of life.

**Table 5:** Factor analysis to estimate factors associated with symptoms.

Symptoms #	Factor 1	Factor 2	Factor 3
1	0.198	0.017	**0.388**
2	**0.700**	−0.046	−0.071
3	**0.704**	0.003	−0.055
4	**0.428**	0.127	0.072
5	−0.118	−0.067	**0.695**
6	**0.771**	−0.040	−0.048
7	**0.521**	0.094	0.009
8	**0.596**	0.011	0.074
9	**0.681**	−0.090	−0.090
10	**0.585**	−0.138	0.129
11	0.303	0.227	0.081
12	0.361	0.350	−0.093
13	0.057	**0.721**	−0.083
14	−0.145	**0.710**	0.027
15	−0.073	0.152	0.133
16	−0.051	−0.050	**0.377**
17	**0.349**	−0.021	0.042
18	−0.047	0.063	**0.367**

Factors that might strongly affect symptoms are shown in bold.

**Fig 2:**
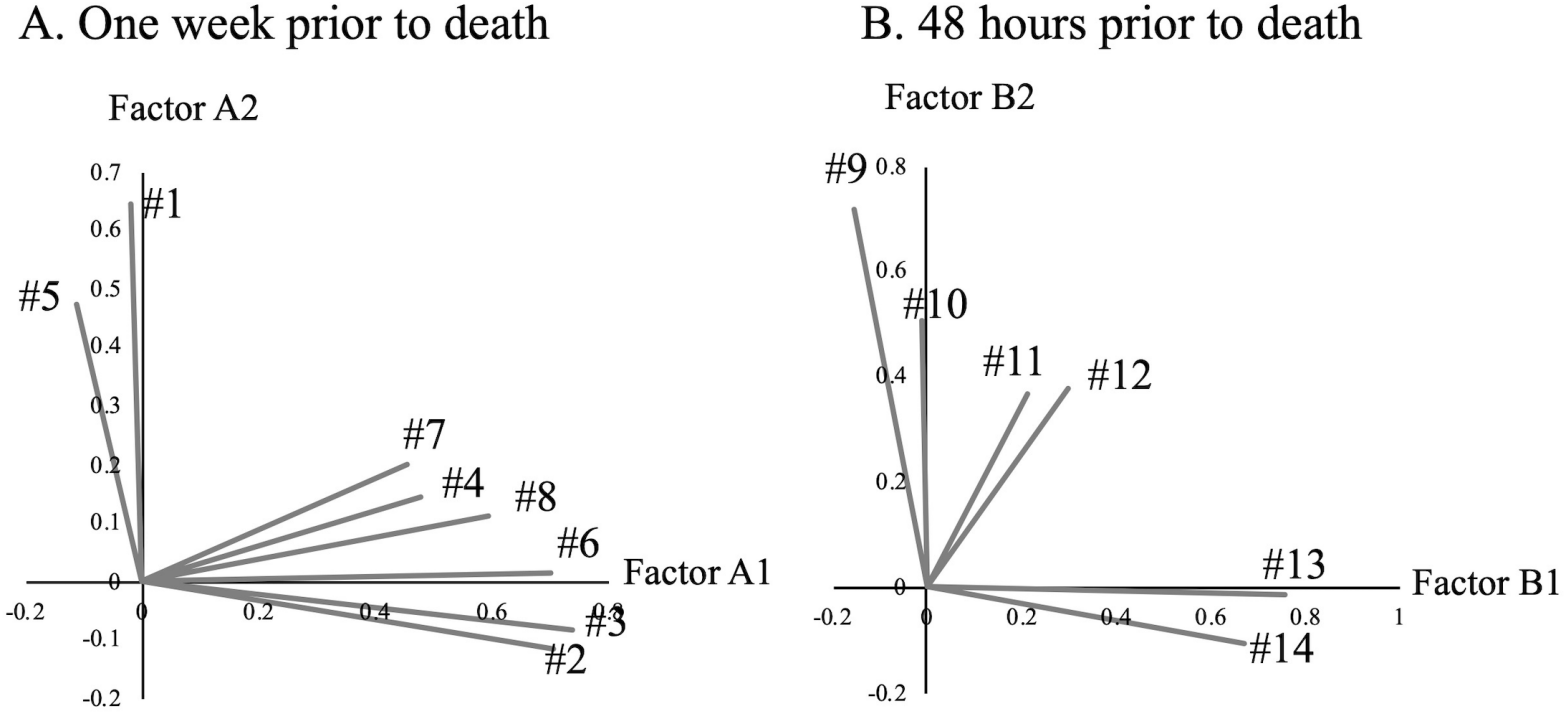
Factor analysis to identify factors associated with symptoms one week prior to death (A) and 48 hours prior to death (B).

Then, we performed a factor analysis on the signs of death observed approximately 1 week before death (1–8; Fig 2A). Two factors (A1 and A2) were identified as appropriate based on eigenvalues >1.0 and the scree plot (S1 Fig). Symptoms 2, 3, 4, 6, 7, and 8 were mainly influenced by factor A1, whereas 1 and 5 were primarily influenced by factor A2. Factor A1 mainly represented global functional decline, while factor A2 mainly represented altered consciousness and agitation. A factor analysis of the signs of death observed approximately 48 h before death was also performed (9–14; Fig 2B). Two factors (B1 and B2) were identified as appropriate using the same criteria. Symptoms 13 and 14 were mainly influenced by factor B1, whereas 9 and 10 were primarily influenced by factor B2. Factor B1 captured peripheral circulatory and skin changes, while factor B2 captured reduced responsiveness and cardiorespiratory compromise. Symptoms 15–18 (observed approximately 12–24 h before death) were too small for factor analysis.

We also performed logistic regression analyses for the signs of death. Pain control and palliative care showed a strong correlation, and both variables had elevated GVIF values (>2). Therefore, only palliative care was included as an explanatory variable in the logistic regression model. Other explanatory variables were age, sex, and place of death (Table 6). In the primary analysis, controlling the false discovery rate (FDR) at 5%, palliative care was significantly associated with symptoms 1–4, 6–14, and 17 (q < 0.05). In a sensitivity analysis using Bonferroni correction, 13 of 18 symptoms remained significant (symptoms 1–4, 6–13, and 17). By place of death, the odds of having symptom 3 were lower in hospitals (adjusted OR 0.438, 95% CI 0.248–0.777) and higher in nursing homes (adjusted OR 4.727, 95% CI 1.594–17.585), compared with home. Both associations were significant after FDR control (hospital, q = 0.020; nursing home, q = 0.029), and the hospital association remained significant after Bonferroni correction. For other covariates that reached nominal significance in the logistic regression, we likewise applied FDR control and Bonferroni correction; none remained significant after adjustment.

**Table 6:** Logistic regression analysis to evaluate factors for each sign of death.

Signs of death	#1. Lower level of consciousness and uttering incoherent statements		#2. Spending extended periods sleeping		#3. Experiencing difficulty consuming meals	
Variable	ORs [95% CI]	*p*-value	ORs [95% CI]	*p*-value	ORs [95% CI]	*p*-value
Sex (female)	0.805 [0.477–1.352]	0.414	1.097 [0.690–1.746]	0.695	0.761 [0.441–1.304]	0.323
Age^†^						
20-64	1.000	–	1.000	–	1.000	–
65-74	0.856 [0.387–1.894]	0.701	0.751 [0.323–1.699]	0.497	1.161 [0.450–2.941]	0.753
75-89	0.596 [0.292–1.236]	0.159	0.888 [0.408–1.856]	0.757	1.288 [0.537–2.974]	0.559
90-	0.678 [0.291–1.565]	0.363	1.072 [0.460–2.437]	0.869	1.368 [0.535–3.406]	0.505
Place of death^††^						
Home	1.000	–	1.000	–	1.000	–
Hospitals	0.868 [0.449–1.608]	0.661	0.617 [0.366–1.047]	0.072	0.438 [0.248–0.777]	0.005*
Nursing homes	0.260 [0.014–1.388]	0.203	0.929 [0.390–2.247]	0.868	4.727 [1.594–17.585]	0.010*
Other places	2.737 [1.000–7.184]	0.043*	1.973 [0.743–5.932]	0.194	3.860 [1.166–17.658]	0.045*
Palliative care	3.395 [1.747–7.053]	<0.001*	4.485 [2.735–7.476]	<0.001*	7.788 [4.449–14.005]	<0.001*
**Signs of death**	**#4. Struggling with drinking, leading to increased risk of aspiration**		**#5. Displaying agitation and heightened limb movement**		**#6. Decreased verbal communication, with diminished auditory clarity**	
**Variable**	**ORs [95% CI]**	***p*-value**	**ORs [95% CI]**	***p*-value**	**ORs [95% CI]**	***p*-value**
Sex (female)	0.719 [0.469–1.095]	0.126	0.665 [0.268–1.619]	0.370	1.148 [0.754–1.751]	0.519
Age^†^						
20-64	1.000	–	1.000	–	1.000	–
65-74	0.915 [0.449–1.862]	0.806	0.245 [0.051–0.909]	0.049*	0.649 [0.316–1.321]	0.235
75-89	0.743 [0.393–1.402]	0.359	0.324 [0.110–0.945]	0.038*	1.274 [0.672–2.408]	0.456
90-	1.251 [0.617–2.551]	0.535	0.297 [0.064–1.117]	0.089	1.253 [0.614–2.558]	0.535
Place of death^††^						
Home	1.000	–	1.000	–	1.000	–
Hospitals	0.631 [0.376–1.044]	0.076	0.542 [0.123–1.689]	0.343	1.295 [0.787–2.144]	0.311
Nursing homes	0.748 [0.302–1.722]	0.516	1.194 [0.059–8.309]	0.877	1.737 [0.744–4.141]	0.204
Other places	2.743 [1.131–7.180]	0.030*	0.984 [0.051–5.829]	0.989	2.134 [0.876–5.510]	0.103
Palliative care	2.097 [1.303–3.422]	0.003*	3.001 [0.878–14.142]	0.111	2.747 [1.712–4.475]	<0.001*
**Signs of death**	**#7. Appearing dazed and devoid of expression**		**#8. Experiencing urinary and fecal incontinence, with inability to reach the bathroom**		**#9. Diminished responsiveness to calls**	
**Variable**	**ORs [95% CI]**	***p*-value**	**ORs [95% CI]**	***p*-value**	**ORs [95% CI]**	***p*-value**
Sex (female)	0.962 [0.631–1.463]	0.855	0.826 [0.536–1.272]	0.386	0.900 [0.574–1.409]	0.647
Age^†^						
20-64	1.000	–	1.000	–	1.000	–
65-74	0.906 [0.445–1.837]	0.784	0.619 [0.298–1.271]	0.194	0.610 [0.271–1.334]	0.222
75-89	0.650 [0.345–1.221]	0.181	1.006 [0.522–1.918]	0.986	0.650 [0.310–1.312]	0.240
90-	0.942 [0.464–1.912]	0.869	0.826 [0.398–1.704]	0.606	1.139 [0.501–2.539]	0.752
Place of death^††^						
Home	1.000	–	1.000	–	1.000	–
Hospitals	1.290 [0.783–2.129]	0.316	0.955 [0.573–1.594]	0.859	1.103 [0.603–1.726]	0.960
Nursing homes	1.849 [0.786–4.361]	0.157	0.748 [0.278–1.888]	0.549	2.036 [0.835–5.274]	0.127
Other places	3.058 [1.270–7.806]	0.015*	1.330 [0.532–3.336]	0.539	1.547 [0.617–4.163]	0.365
Palliative care	2.498 [1.549–4.098]	<0.001*	4.082 [2.517–6.738]	<0.001*	4.166 [2.547–6.940]	<0.001*
**Signs of death**	**#10. Impaired speech clarity**		**#11. Exhibiting distress due to shallow breathing**		**#12. Difficulty in detecting pulse or experiencing a drop in blood pressure**	
**Variable**	**ORs [95% CI]**	***p*-value**	**ORs [95% CI]**	***p*-value**	**ORs [95% CI]**	***p*-value**
Sex (female)	0.722 [0.468–1.111]	0.139	0.866 [0.563–1.330]	0.510	1.184 [0.773–1.818]	0.437
Age^†^						
20-64	1.000	–	1.000	–	1.000	–
65-74	0.880 [0.422–1.822]	0.730	0.932 [0.456–1.902]	0.846	0.736 [0.359–1.500]	0.400
75-89	0.637 [0.329–1.212]	0.173	1.008 [0.534–1.902]	0.980	0.796 [0.422–1.499]	0.478
90-	0.580 [0.278–1.195]	0.142	0.908 [0.442–1.863]	0.791	0.839 [0.411–1.712]	0.630
Place of death^††^						
Home	1.000	–	1.000	–	1.000	–
Hospitals	0.731 [0.436–1.219]	0.231	1.112 [0.666–1.854]	0.684	1.012 [0.607–1.676]	0.962
Nursing homes	0.685 [0.244–1.752]	0.445	1.015 [0.377–2.567]	0.975	0.832 [0.315–2.044]	0.697
Other places	0.855 [0.332–2.113]	0.738	0.870 [0.325–2.191]	0.772	0.708 [0.258–1.768]	0.476
Palliative care	3.314 [2.047–5.445]	<0.001*	3.682 [2.245–6.171]	<0.001*	2.407 [1.476–3.991]	<0.001*
**Signs of death**	**#13. Limbs becoming cold**		**#14. Development of cyanosis on the face or extremities**		**#15. Sensation of warmth and reluctance to wear clothing**	
**Variable**	**ORs [95% CI]**	***p*-value**	**ORs [95% CI]**	***p*-value**	**ORs [95% CI]**	***p*-value**
Sex (female)	0.777 [0.497–1.211]	0.265	1.139 [0.687–1.894]	0.614	0.393 [0.108–1.251]	0.128
Age^†^						
20-64	1.000	–	1.000	–	1.000	–
65-74	0.960 [0.459–2.012]	0.914	0.750 [0.327–1.709]	0.493	0.704 [0.173–2.835]	0.615
75-89	0.980 [0.512–1.895]	0.951	0.769 [0.378–1.599]	0.474	0.268 [0.063–1.114]	0.066
90-	1.017 [0.484–2.146]	0.965	0.786 [0.344–1.797]	0.566	n.e. [n.e.}	–
Place of death^††^						
Home	1.000	–	1.000	–	1.000	–
Hospitals	0.564 [0.316–0.978]	0.046*	0.725 [0.367–1.356]	0.331	0.188 [0.010–1.020]	0.117
Nursing homes	0.948 [0.337–2.445]	0.915	2.120 [0.768–5.559]	0.133	n.e. [n.e.}	–
Other places	1.281 [0.498–3.159]	0.595	1.134 [0.351–3.120]	0.818	n.e. [n.e.}	–
Palliative care	2.913 [1.726–5.044]	<0.001*	2.349 [1.274–4.517]	0.008*	1.254 [0.302–8.539]	0.780
**Signs of death**	**#16. Experiencing agitation or delirium**		**#17. Slight limb movements despite being bedridden**		**#18. Tremors or shivering of coverings draped over the body**	
**Variable**	**ORs [95% CI]**	***p*-value**	**ORs [95% CI]**	***p*-value**	**ORs [95% CI]**	***p*-value**
Sex (female)	1.320 [0.519–3.463]	0.563	0.939 [0.590–1.493]	0.789	0.536 [0.260–1.074]	0.083
Age^†^						
20-64	1.000	–	1.000	–	1.000	–
65-74	0.622 [0.168–2.157]	0.457	1.267 [0.587–2.768]	0.549	0.685 [0.242–1.906]	0.468
75-89	0.369 [0.110–1.218]	0.098	1.174 [0.593–2.385]	0.650	0.555 [0.225–1.413]	0.205
90-	0.196 [0.031–0.929]	0.055	1.577 [0.731–3.469]	0.250	0.404 [0.116–1.286]	0.135
Place of death^††^						
Home	1.000	–	1.000	–	1.000	–
Hospitals	1.371 [0.421–3.859]	0.569	0.998 [0.567–1.722]	0.995	0.337 [0.097–0.895]	0.049*
Nursing homes	2.472 [0.117–19.333]	0.445	0.948 [0.316–2.541]	0.919	0.608 [0.032–3.636]	0.649
Other places	4.110 [0.558–20.206]	0.105	0.712 [0.222–1.938]	0.531	1.002 [0.150–4.000]	0.998
Palliative care	2.828 [0.803–13.622]	0.139	3.083 [1.770–5.556]	<0.001*	2.476 [1.004–7.093]	0.065

† Those aged 20–64 = 1.

†† Home = 1.

CI: confidence interval.

* donates nominal *p* < 0.05. Statistical significance for the primary hypotheses was determined by controlling the Benjamini–Hochberg FDR at 5% across the 18 symptom-wise tests; Bonferroni-corrected results are provided as a sensitivity analysis in the text.

n.e.= not estimable. The odds ratio and 95% confidence interval could not be calculated because the event count in this category was extremely low or zero, resulting in quasi-complete separation in the logistic regression model.

## Discussion

### Principal findings

This study identified disparities in patient characteristics by place of death and age at death. The ORs for pain control and palliative care were significantly lower in the older age groups. The ORs for pain control and palliative care were also lower in nursing homes compared with those at home. The ORs for having living wills were significantly lower in patients aged ≥90 years than in those aged 20–64 and in hospitals than at home. In addition, we conducted factor analyses of various signs of death and identified three latent factors (Table 5). Furthermore, we found that palliative care was independently associated with higher odds of signs loading on factor 1.

### Strengths and Limitations

The strengths of this study lie in simultaneously comparing multiple places of death (home, hospital, and nursing home) within a unified framework while assessing core care indicators—pain control, palliative care, and the presence of living wills. Using logistic regression adjusted for age, sex, and place of death, we estimated associations that are not readily captured by simple tabulations. Moreover, by organizing 18 signs of death into three factors, we demonstrated an association between palliative care and the signs represented by factor 1 (global terminal decline), providing a new perspective for the care of patients at the end of life.

This study has several limitations. First, selection bias is possible. Only physicians affiliated with JSDD, who may have a particular interest in end-of-life care, were included; therefore, their responses to the questionnaire might differ from those observed in general end-of-life care. The questionnaire explicitly instructed respondents to “answer about one patient you remember among those you cared for at the end of life within the past five years,” which could introduce recall and salience biases, favoring especially memorable cases for reasons unknown to the investigators. As a result, the observed frequencies and constellations of signs may overrepresent prominent or unusual presentations and may not fully reflect typical trajectories. Second, responses may be inaccurate if based solely on memory, because whether participants consulted medical records or relied only on recollection was left to their discretion. Third, the questionnaire asked whether both pain control and palliative care were present or absent, but we did not provide operational definitions of these terms; therefore, each participant may have answered according to their own understanding. Consequently, misclassification is possible, including differential misclassification across settings, which may have biased the associations. Fourth, we did not collect information on the responding physician’s practice setting nor on when they treated the reported patient or whether their involvement covered the full end-of-life course; moreover, because physicians could select a patient from the preceding five years and recruitment spanned 20 months, the reported cases likely occurred over more than six years, during which end-of-life care practices may have changed. Fifth, although the questionnaire was developed with input from physicians with expertise and clinical experience in end-of-life care, we did not conduct a formal instrument development process. In addition, no pretest was performed. Therefore, the content validity and measurement properties of some items may be limited and could have introduced misclassification. Finally, because this study lacked data on the cause of death, the associations between patients’ underlying conditions and the care they received and the presence of a living will remain unknown.

### Interpretation

In the present study, patients’ age and place of death were significantly associated with the provision of pain control and palliative care. In addition, Spearman’s correlation coefficient between pain control and palliative care was significant, indicating a tendency for both to be provided concurrently (Fig 1B). Adequate pain control is a fundamental component of palliative care and, along with symptom management, is considered essential. Given the close relationship between pain control and palliative care, it is reasonable that similar results were obtained for both. The differences in the implementation rates of these forms of care by age might stem from variations in the underlying causes of death, with people aged <65 years more likely to die from cancer, whereas those aged >90 years are more likely to die from dementia and senility. However, it should be noted that there is considerable heterogeneity among older adults at the end of life. For example, 86% of the individuals who died in nursing homes experienced pain during their final 3 months [32]. In addition, patients with terminal dementia experienced distress as death approached, with 63.4% and 29.6% of them experiencing high and intermediate levels of suffering, respectively [33]. A study employing standardized questionnaires (symptom management in end-of-life with dementia) among patients at the end-of-life revealed no significant differences in scores among patients with cancer, chronic diseases, and dementia in the last month of life; however, patients with dementia had higher levels of pain, and those with cancer exhibited lower levels of anxiety [34]. A study comparing end-of-life symptoms between patients with young-onset and late-onset dementia found similar circumstances of death, symptoms, and treatment between the two groups [35]. Thus, suffering appears to be prevalent among older adults nearing death regardless of their underlying conditions. Therefore, it is important to provide pain control or palliative care as needed, guided by a comprehensive geriatric assessment.

In the present study, more patients received palliative care at home than in other places. Our data are consistent with a previous review showing that factors associated with home death include multidisciplinary home palliative care, a preference for home death, a cancer diagnosis as opposed to other diagnoses, and not living alone [36]. Since home-based palliative care might be cost-effective and reduce acute hospital admissions [37,38], this represents a favorable situation. In contrast, the proportion of patients receiving pain management or palliative care in nursing homes was lower. This suggests challenges in achieving pain control and palliative care in nursing homes. In Canada, various diseases are considered indications for palliative care, and palliative care is widely provided to people with dementia [39]. In Japan, palliative care is primarily targeted toward patients with cancer [40], and due to restrictions in the medical insurance system, there are insufficient hospices or palliative care units where patients with non-cancer illnesses can receive such care [41]. Therefore, it is important to establish a system that enables older adults with non-cancer conditions to have better access to palliative care, whether they are in hospitals or nursing homes.

In the present study, 41.3% of patients had a documented living will before death. This percentage was comparable to previously reported ACP implementation rates in Japan [9]. The prevalence of having a living will was significantly lower among patients who died in hospitals, while there was no significant difference between those who died at home and those who died in nursing homes (Table 4). In contrast, an Australian study reported that patients at home had end-of-life discussions more frequently than patients living in residential aged care facilities [25]. Our findings suggest that end-of-life care experts may facilitate living-will documentation in nursing homes as frequently as home. ACP may be influenced more strongly by the usual place of living than by the place of death itself. Patients who die in hospitals constitute a heterogeneous group, including community-dwelling persons transferred for acute deterioration and residents of nursing homes admitted shortly before death. Consequently, differences by place of death may partly reflect prior access to ACP in the regular living setting rather than hospital practices per se; therefore, these results should be interpreted with caution. Future studies should adjust for usual residence, admission source, length of stay, and the timing of living-will documentation. Even among patients receiving care from end-of-life experts, the proportion with a living will was significantly lower in those aged ≥90 years (Table 4). Although older adults living at home tended to avoid contemplating possible complications and delegate end-of-life decisions to others, their decisions tended to change with age and declining health status [42]. In addition, ACP has the potential to mitigate emergency department visits and ambulance calls among patients residing in nursing homes [43]. Therefore, practicing ACP, including obtaining a living will, at a younger age might be recommended.

Factor analysis simplifies a set of complex items using statistical procedures to explore the underlying dimensions that explain the relationships between the multiple items [44]. In the present study, factor 1 reflects global terminal decline (neurocognitive–functional deterioration), with high loadings for decreased consciousness and communication, poor oral intake, incontinence, and diminished responsiveness. Factor 2 captures peripheral circulatory and skin changes, including cold extremities and cyanosis. Factor 3 represents terminal agitation or delirium with motor restlessness, including agitation, incoherent utterances, delirium-like behaviors, and shivering of coverings. Furthermore, upon comparison of Table 5 with Fig 2, factors A1 and A2 observed 1 week before death were considered to correspond to factors 1 and 3, respectively, in the overall signs of death, whereas factors B1 and B2 observed 48 h before death corresponded to factors 2 and 1, respectively. Despite the diverse nature of death signs observed at various intervals, they were suggested to be influenced by shared underlying factors. The signs mainly associated with factor 1 were generally common, whereas those mainly linked to factor 3 were infrequent (Table 2). Thus, factor 1 was considered the dominant factor in the signs of death. Factor analyses have been performed on dying patients [45]; a study revealed four aspects of death anxiety: dysphoria, death intrusion, fear of death, and avoidance of death [46]. To the best of our knowledge, this is the first study to perform a factor analysis on various signs of death, and might lead to a structural explanation of them.

In the logistic regression analysis, palliative care was independently and significantly associated with multiple signs of death (Table 6). Many of the signs associated with factor 1 (global terminal decline) in Table 5 were more frequently observed in patients receiving palliative care. In contrast, among the signs associated with factor 3 (agitation or delirium), only symptom 1 (lower level of consciousness and uttering incoherent statements) showed a significant increase in the palliative care group. It is plausible that the use of sedatives as part of palliative care may contribute to signs related to decreased responsiveness and reduced activity. However, considering that palliative care is often introduced in situations involving severe suffering, this association may reflect correlation rather than causation. Moreover, since palliative care in Japan is predominantly provided to patients with cancer and less frequently to those with dementia, differences in underlying causes of death may also have influenced the observed patterns of signs.

The OR for symptom 3 (experiencing difficulty consuming meals) was lower in hospitals and higher in nursing homes, compared with home (Table 6). Hospitalized patients do not necessarily receive oral nutrition, as enteral or intravenous feeding is sometimes provided. In contrast, many nursing home residents consume food orally, but they often have higher care dependency and a higher prevalence of dementia. These setting-specific differences in patient characteristics may have contributed to the variation in the observed frequency of symptom 3.

### Generalizability

This study is subject to potential bias because the included cases were end-of-life patients cared for by physicians registered with the JSDD, and responses were based on physicians’ recollection, with the reported cases likely representing “memorable” patients. Therefore, our findings cannot be readily generalized to the broader population of deceased patients. Representative studies recruiting a wider range of end-of-life patients are needed. Because the cause of death is also clinically important, future studies should collect information on underlying diseases and incorporate these variables into the analyses. Nonetheless, examining the structure of signs of death through factor analysis in this study may help strengthen the questionnaire in future work. The conceptual framework suggested by our results—potential care disparities by age and place of death and the latent structure of signs of death—can be tested in other populations and may help inform service planning across care settings.

## Conclusions

This study suggests that the implementation of pain control, palliative care, and living wills differs by age group and place of death in a physician-recalled sample of end-of-life patients. Signs of death clustered into three factors, and signs reflecting global terminal decline were associated with receipt of palliative care. These findings highlight potential targets for improving end-of-life care across settings and support the need for representative, prospective studies to confirm these associations and inform service planning.

## Supporting information

S1 Fig Scree plots for exploratory factor analyses of signs of death. Scree plots show eigenvalues by factor number for the exploratory factor analyses conducted on the signs of death assessed in this study. Panel A displays the analysis including all 18 signs (symptoms 1–18), Panel B includes the eight signs assessed approximately 1 week before death (symptoms 1–8), and Panel C includes the six signs assessed approximately 48 hours before death (symptoms 9–14).
(TIFF)
