# Supplementary figures and images for: The characteristics of patients’ medical care, living will, and signs of death by age and the place of death: A cross-sectional study using a questionnaire survey targeting physicians with expertise in end-of-life care
Source: PLoS One. 2026 Mar 24;21(3):e0343868. doi: 10.1371/journal.pone.0343868 (PMC13012453; doi:10.1371/journal.pone.0343868)

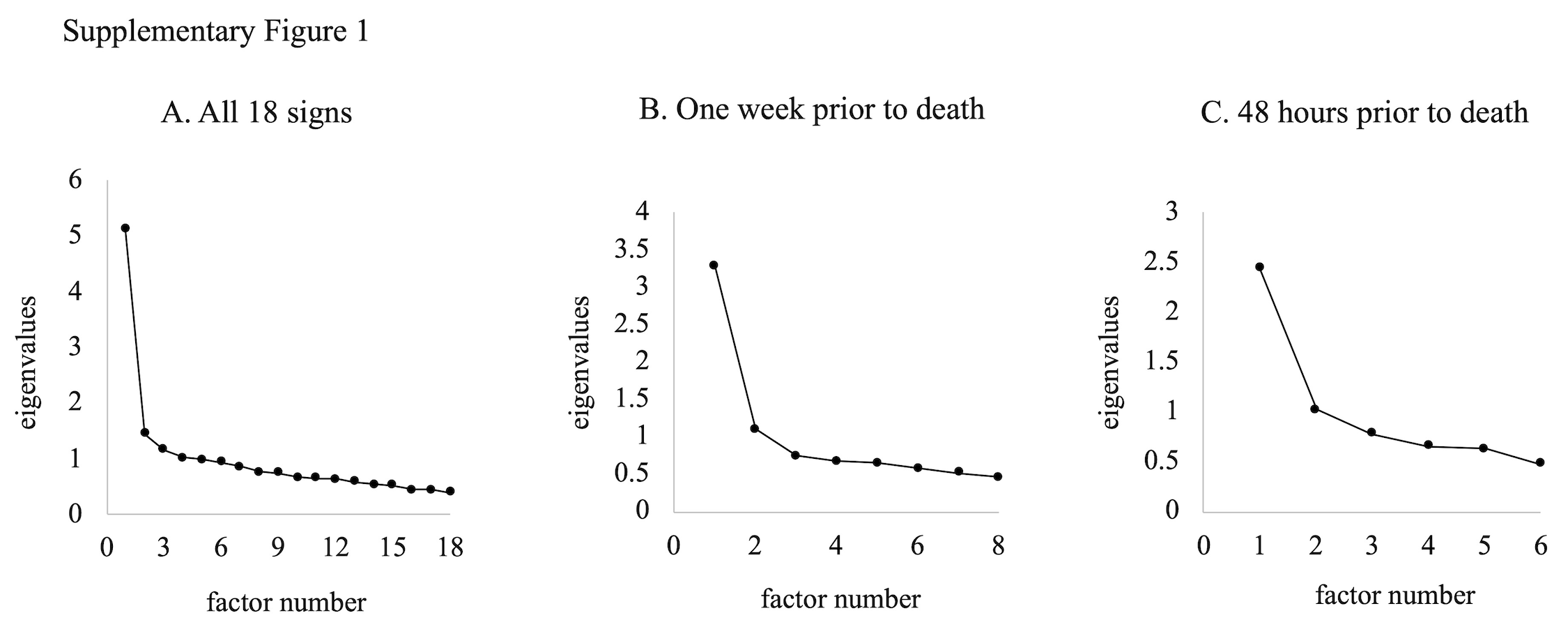

Supplement: S1 Fig — Scree plots show eigenvalues by factor number for the exploratory factor analyses conducted on the signs of death assessed in this study. Panel A displays the analysis including all 18 signs (symptoms 1–18), Panel B includes the eight signs assessed approximately 1 week before death (symptoms 1–8), and Panel C includes the six signs assessed approximately 48 hours before death (symptoms 9–14). (TIFF) [file pone.0343868.s001.tiff]
